# Measuring the impact of spatial perturbations on the relationship between data privacy and validity of descriptive statistics

**DOI:** 10.1186/s12942-020-00256-8

**Published:** 2021-01-07

**Authors:** Kelly Broen, Rob Trangucci, Jon Zelner

**Affiliations:** 1 Department of Epidemiology, University of Michigan School of Public Health, Ann Arbor, MI 48109 USA; 2 Center for Social Epidemiology and Population Health, University of Michigan School of Public Health, Ann Arbor, MI 48109 USA; 3 Dept. of Statistics, University of Michigan, Ann Arbor, MI 48109 USA

**Keywords:** Geomasking, Privacy, Spatial anonymity, Reproducibility

## Abstract

**Background:**

Like many scientific fields, epidemiology is addressing issues of research reproducibility. Spatial epidemiology, which often uses the inherently identifiable variable of participant address, must balance reproducibility with participant privacy. In this study, we assess the impact of several different data perturbation methods on key spatial statistics and patient privacy.

**Methods:**

We analyzed the impact of perturbation on spatial patterns in the full set of address-level mortality data from Lawrence, MA during the period from 1911 to 1913. The original death locations were perturbed using seven different published approaches to stochastic and deterministic spatial data anonymization. Key spatial descriptive statistics were calculated for each perturbation, including changes in spatial pattern center, Global Moran’s I, Local Moran’s I, distance to the k-th nearest neighbors, and the L-function (a normalized form of Ripley’s K). A spatially adapted form of k-anonymity was used to measure the privacy protection conferred by each method, and its compliance with HIPAA and GDPR privacy standards.

**Results:**

Random perturbation at 50 m, donut masking between 5 and 50 m, and Voronoi masking maintain the validity of descriptive spatial statistics better than other perturbations. Grid center masking with both 100 × 100 and 250 × 250 m cells led to large changes in descriptive spatial statistics. None of the perturbation methods adhered to the HIPAA standard that all points have a k-anonymity > 10. All other perturbation methods employed had at least 265 points, or over 6%, not adhering to the HIPAA standard.

**Conclusions:**

Using the set of published perturbation methods applied in this analysis, HIPAA and GDPR compliant de-identification was not compatible with maintaining key spatial patterns as measured by our chosen summary statistics. Further research should investigate alternate methods to balancing tradeoffs between spatial data privacy and preservation of key patterns in public health data that are of scientific and medical importance.

## Background

Researchers in public health, medicine, and the social sciences are facing a reproducibility crisis that continues to grow with the complexity of data collection, cleaning, and analysis pipelines. A reproducible study has been defined broadly as one from which a researcher can duplicate results using the data from the original analysis and the methods described in the study [1]. To meet these standards, many peer-reviewed journals are implementing policies to increase data transparency and public availability. In practice, meeting this standard can prove to be quite difficult. These issues are magnified in public health and medicine, where ethical and legal protections of patient and research subject privacy must be considered ahead of the public health and scientific benefits of reproducibility. These issues are particularly acute for spatially referenced disease and health data which may reveal not only the identity but the spatial location of individuals with sensitive health conditions, e.g. HIV infection, or behavioral risks such as injection drug use [2]. These roadblocks to a consistently reproducible spatial epidemiology have limited the application of powerful spatiotemporal analytic tools in public health practice. This represents a significant loss to public health, as such data can provide insights into how to best intervene on a wide range of health conditions, ranging from those associated with exposure to environmental toxicants, spatially concentrated social inequality, and infectious disease transmission [3–6].

For example, as recent work in the area of vaccine-preventable diseases has shown, the scale at which such data are reported can determine the nature and the quality of inferences that can be drawn from them [7]. In recent months, the COVID-19 pandemic has shown the crucial role of understanding the determinants of fine-scale spatial variation in infection outcomes, as such data are key for understanding differential risks of mortality by age, socioeconomic status and as a function of neighborhood environments. This has created an unprecedented amount of interest in making individual level case data publicly available, with multiple sources producing maps of case and testing rates [8–11]. As analysts produce maps for public release in the rapidly changing pandemic setting, maintaining individuals’ privacy is increasingly essential as stigma-driven harassment also increases [12, 13]. While all maps are using aggregated counts, the level to which data has been aggregated varies; some maps are providing data at as low a level as the zip code level while many only release information by county [8–11]. More granular maps have suppressed data for zip codes with limited numbers of cases, but there are no standardized limits for data release [10].

A number of geomasking methods have been proposed to address the problem of identifiability in publicly released spatial health data. Geomasking algorithms shift the coordinates of a point of interest in a way that is intended to reduce the likelihood of identification of all individuals in the dataset to the point that it no longer presents a meaningful risk of identification. However, there has been relatively little attention paid to the amount of spatial information lost relative to privacy protection gained from each of these approaches. In this paper, we measured the tradeoff between increased privacy and spatial information loss provided by a wide variety of geomasking approaches applied to the same detailed dataset. We used an array of geographic perturbation methods described in the literature on spatial analysis and medical geography, which are commonly employed in the public release of sensitive spatial data, as well as a widely-used metric of anonymization known as *k-anonymity* [14].

A better understanding of the nature and extent of these tradeoffs is necessary to allow researchers, regulatory bodies such as IRBs, and data providers such as public health departments and hospitals, agree on spatial perturbation methods that can preserve patient or participant privacy, while understanding how they may result in potential biases that could limit the utility of such data for different types of analyses.

The acceptable ratio of information lost to privacy gain is likely to vary as a function of (1) the sensitivity of the underlying data, (2) the nature of the data sharing, e.g. with a trusted partner subject to a data use agreement vs. wide public release, and (3) the public health urgency of the problem the data may aid in solving. These questions have always been pertinent, but the COVID-19 pandemic has forced them towards the front of the conversation.

### Privacy-first reproducibility

A commonly discussed standard for reproducibility in public health and medicine is that published analyses should include access to all underlying data, the exact methods employed from data processing to analysis and figure generation (including the code to run all analyses), and documentation sufficient to run the provided code on the provided data and obtain the published results [15]. Finally, all of these components should be distributed in a way that makes them widely accessible (e.g. under a permissive software license, hosted on an open and visible platform such as *github*) [15]. Done properly, this allows others to directly validate results, rapidly deploy new methods and pursue alternate hypotheses using the original data [16]. However, this maximally transparent approach is ethically and legally prohibited when the relevant data contain identifiable information including home addresses and key patient demographics. These are considered protected health information (PHI) under the Health Insurance Portability and Accountability Act (HIPAA) in the United States, and the General Data Protection Regulation (GDPR) in the European Union. Therefore, this data cannot be publicly released in an unmasked form [17]. While other countries have implemented protection measures for individual’s privacy, a 2016 update of the GDPR made it one of the strongest data protection laws, so methods that comply with the GDPR will likely comply with other protection policies [18]. In this paper, we argue for and outline the contours of a *privacy first* approach to reproducibility that balances these ethical and legal obligations to individuals with potential benefits to public health. While results may not be completely replicable (in which the exact same results are obtained), they can be reproducible (the same methods can be applied and results are similar) and data can be transparently submitted as the results are peer-reviewed. Although the HIPAA statute an GDPR do not lay out specific standards for what constitutes an unacceptable level of identifiability, a common interpretation of HIPAA requirements on data release is that each data point must be indistinguishable from at least 10 others in the same dataset [19].

Under HIPAA and GDPR, data may be released after all identifiable information is removed; under HIPPA, this refers to 18 specific attributes, whereas under the GDPR it means any information that may lead to the direct or indirect identification of a person [17, 18]. The unit of interest in geospatial epidemiology—an individual’s location or set of locations visited over time—is clearly sensitive, identifiable information, and therefore methods for deidentification of spatial data must be robust to malicious reverse engineering. Despite the importance of these methods for completing privacy-respecting reproducible research, little is known about how to leverage different methods of spatial perturbation to accomplish the twin goals of (1) maximizing participant privacy (i.e. minimizing identifiability) while (2) maintaining key spatial patterns necessary for reproducibility and verification of published results [20]. Because of this lack of guidance on how to best de-identify individual-level spatial health data to maintain compliance, spatial epidemiologists and other health researchers face significant barriers to reproducibility. HIPAA outlines two approaches by which de-identification can be considered to have been achieved:

1. Safe harbor: This method requires the removal of all identifiers. Only the first three digits of zip codes are maintained if “the geographic unit formed by combining all ZIP codes with the same three initial digits contains more than 20,000 people”. If the geographic unit contains 20,000 or fewer people, all five digits of the ZIP code are removed [21].
2. Expert determination: Under this approach, “a person with appropriate knowledge of and experience with generally accepted statistical and scientific principles and methods for rendering information not individually identifiable” implements a scientifically verified method on spatially identifiable data until there is “very small risk that [the] intended recipient could identify [the] individual” [17]. Although HIPAA does not explicitly quantify this risk, it is commonly interpreted as each individual being indistinguishable from at least 9 other individuals in the dataset [19].

The GDPR employs criteria similar to expert determination, stating that anonymous data is no longer protected and anonymity is achieved when the data is manipulated in a manner by which it could not be re-identified by “all the means likely reasonably to be used” [18]. Like HIPAA, this does not provide a single metric of spatial anonymity. Despite efforts to develop geomasking methods that can meet these standards, there is no consensus on how to choose an approach. Previous work in this area has tested only one or a small number of perturbation approaches at a time [22–24], making comparison to other perturbation methods infeasible. The primary measure of privacy employed by these studies is k-anonymity. However, the implementation of this metric across studies has been inconsistent [22–24]. In this study, we implemented seven perturbation methods using a single dataset, and we compared outcomes using a k-anonymity metric appropriate to our data, in which only deaths are geocoded.

## Methods

### Data

We geocoded the household location of each of the 4050 deaths recorded in Lawrence, Massachusetts from 1911 to 1913. We used a historical dataset so that the underlying data can be released while complying with HIPAA and GDPR standards, as all individuals have been deceased for > 50 years [17]. We used ArcGIS Version 10.6.1 to create a complete map of the city limits from a set of historical maps. Each address in the death register was located and geocoded using the original maps. Shapefiles for boundaries of the city of Lawrence, Massachusetts, and the Merrimack River were obtained from Mass.gov [25, 26].

### Analysis

We employed seven different perturbation methods, which were selected to capture the range of approaches that are useful and feasible with case-only data, as compared to case–control data. We examined both non-aggregating perturbations, which move points to unique locations, and aggregating perturbations, which agglomerate points into a single location.

#### Non-aggregating perturbations

1. Random perturbation: Each case is moved a randomly selected distance in a randomly selected direction. Perturbed locations are not restricted to the bounds of the study area, but two maximum perturbation distances were employed, restricting points to locations within a 50- or 250-m radius. These radii were selected because each point has on average approximately 12 points within 50 m of it and 182 within 250 m of it, so moving the points these distances could potentially have as many as 11 and 181 points closer to the original point than the perturbed point (Fig. 1).

**Fig. 1:**
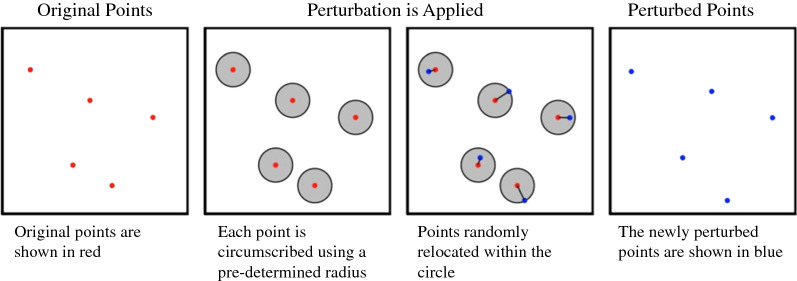
Visual representation of random perturbation
2. Random weighted perturbation: Same as random perturbation, but the maximum distance for each case is constrained to the distance to the point’s k-th nearest neighbor. We implemented random weighted perturbation twice, with points moved within the distance of the 5th and 50th nearest neighbors. These k-th neighbor values were selected to test different levels of anonymity since moving a point the distance to its k-th nearest neighbor means it could potentially have as many as k-1 points closer to the original point than the perturbed point.
3. Donut masking: Each case is moved in a random direction within a random distance constrained to an interval defining a maximum and minimum distance [27]. Donut masking was implemented twice, with points moved between 5–50 m and 50–250 m. These distances were chosen because they moves points between the 1st and 5th nearest neighbors and 5th and 100th nearest neighbors, respectively (Fig. 2).

**Fig. 2:**
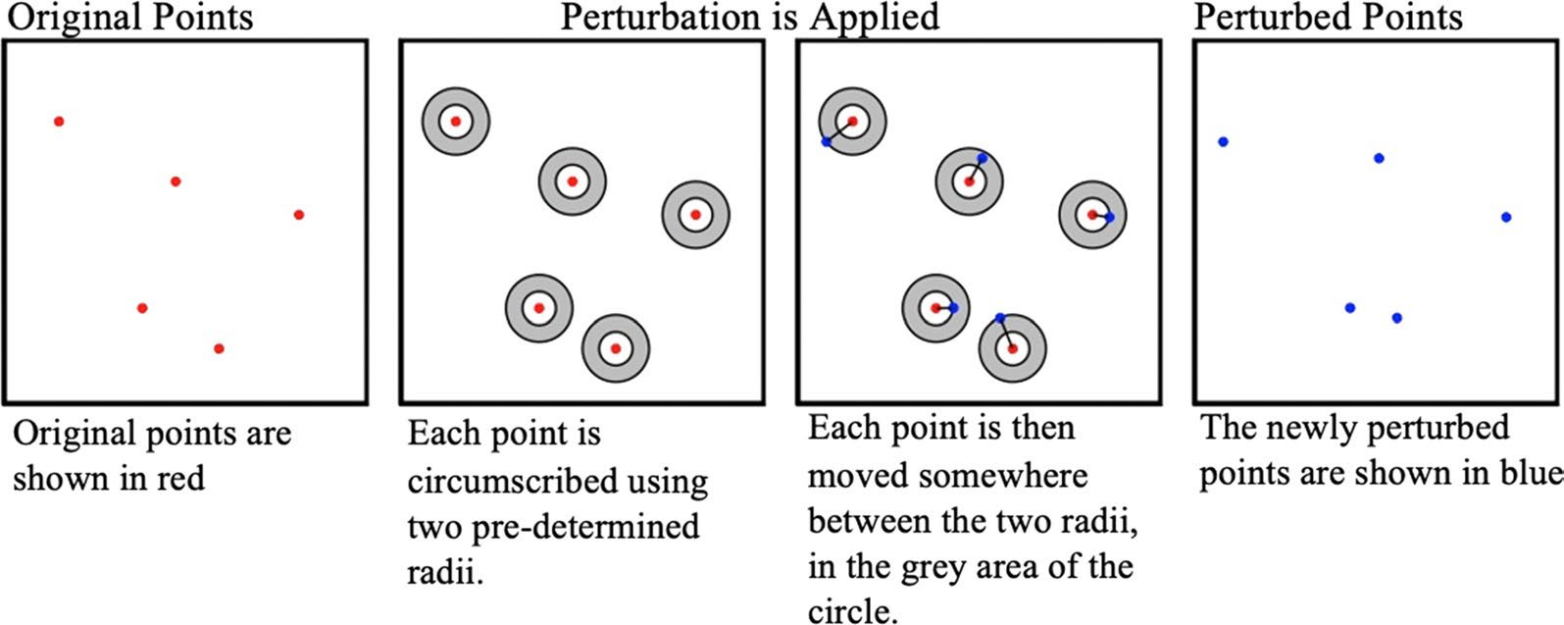
Visual representation of donut masking
4. Horizontal shear: Cases are perturbed using a linear transformation to shear the data horizontally. We shifted each point along its x axis until it was 45° away from its original position relative to the center of the distribution of points [24] (Fig. 3).

**Fig. 3:**
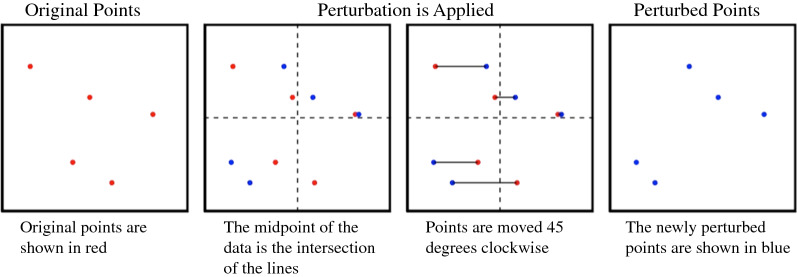
Visual representation of horizontal shear
5. Voronoi masking: This approach moves each case to a point on the nearest edge of its Voronoi tessellation, or the polygon around the original points where the lines are equidistant to the point and its nearest points [28]. Although Voronoi masking does not always move points together, if two points are both each other’s nearest neighbor, they will be snapped together so Voronoi masking does have some degree of aggregating effect (Fig. 4).

**Fig. 4:**
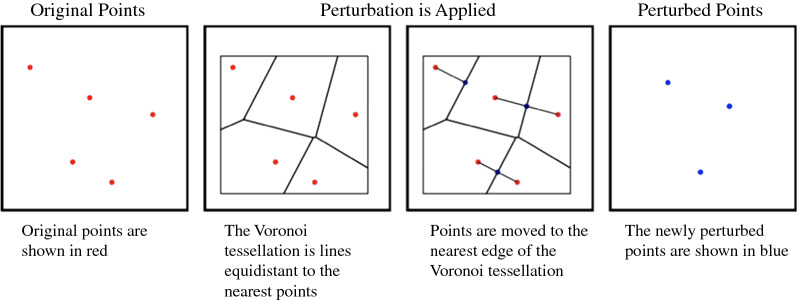
Visual representation of Voronoi masking

#### Aggregating perturbations

Aggregating perturbations move multiple points to the same centroid of a cell within a user-defined grid, effectively hiding the individual within a larger population [29]. We employed two methods of aggregation adapted from Seidl et al. [22]:

1. Grid line masking: Points are moved to the nearest edge of their enclosing grid cell (Fig. 5).

**Fig. 5:**
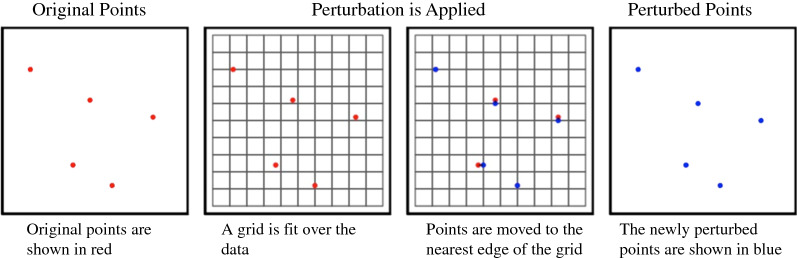
Visual representation of grid line masking
2. Grid center masking: Points are moved to the centroid of the cell within which they are located (Fig. 6).

**Fig. 6:**
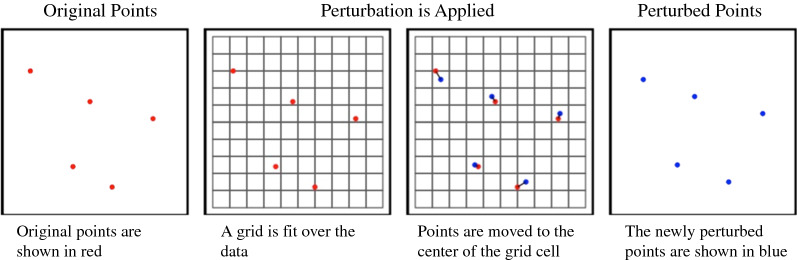
Visual representation of grid center masking

To understand how the resolution of the grid employed impacts our outcomes, both of these were performed using a fine-scale grid (100 m × 100 m, or roughly the average distance to the 20th nearest neighbor) and a coarser one (250 m × 250 m, or roughly the average distance to the 100th nearest neighbor).

## Spatial measures

To determine how much and which types of information were preserved by each approach, we compared each perturbed dataset to the original data using multiple spatial statistics:

1. Point center: The center of the spatial distribution is calculated as the mean and median of the point coordinates, comparing each perturbation to the original data. The difference in mean and median from the unperturbed data was calculated as the Euclidean distance between the points. Changes in the center of the spatial distribution demonstrate the overall movement of points resulting from each perturbation.
2. Global Moran’s I: This is a measure of spatial clustering ranging from − 1 (complete separation) to 1 (complete clustering) [30]. Points were aggregated to 200 × 200 m cells and Global Moran’s I was calculated to compare if the number of deaths in a cell is overall similar or dissimilar to the number of deaths in surrounding cells.
3. Local Moran’s I: This is a measure of local spatial autocorrelation, indicating how similar a spatial unit is to its surrounding neighbors. As with Global Moran’s I, values range from [− 1, 1] [31]. As with Global Moran’s I, points were aggregated to 200 × 200 m cells.
4. Distance to Kth-nearest Neighbor: For each perturbation, the average distance of a death to its 1st, 5th, 10th, and 20th neighbors was calculated and compared to the same distance in the unperturbed data as in [22]. As points become more clustered in space, average distance to the kth nearest neighbor decreases. Examining the 1st, 5th, 10th, and 20th neighbor allows us to measure the magnitude of clustering or dispersion conferred by a perturbation.
5. L-Function: The last spatial metric computed is the L-function, a normalized form of Ripley’s *K*. The L-function calculates the expected number of points within a multi-dimensional ball o f radius *r*, divided by the volume of the ball [32]. This is used to assess whether the points within a fixed distance of a given location demonstrate clustering or repulsion to an extent greater than would be expected by random chance alone.

## Measuring de-identification

**Fig. 7:**
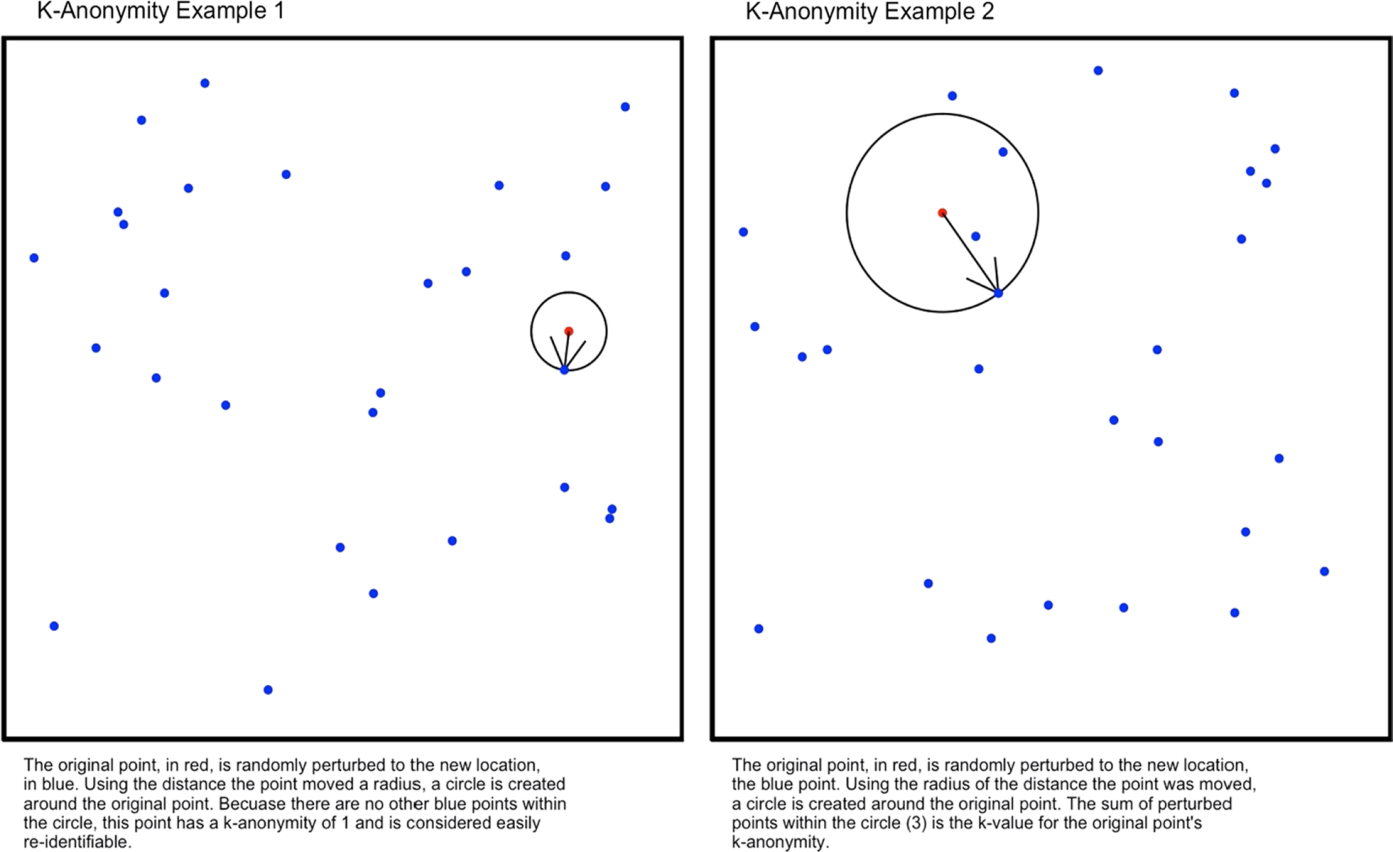
Visual representation of K-anonymity using only the geocoded points and no underlying population data

We used k-anonymity, which is a metric widely used to measure the degree of privacy conferred by a particular perturbation. Specifically, in a dataset with a k-anonymity of 10, each released record is indistinct from at least 9 (k − 1) other records [14]. For non-spatial data, this typically requires deleting or randomizing data fields until there are at least *k* − 1 indistinct records for each case. In the context of spatial data, k-anonymity refers to the number of perturbed points closer to the unperturbed point than its own perturbation. An individual point’s k-anonymity is measured using the number of newly perturbed points that fall within a circle around the point’s new, perturbed location, with the radius of that circle equal to the distance the point was moved by the perturbation [23]. When using datasets that include locations of non-case data, these can be included in the k-anonymity measure as points the case is indistinguishable from; since our data did not include non-case data, this interpretation of K-anonymity was used. K-anonymity is typically reported as both the average k across each point in the dataset, as well as the minimum k. To ensure protection for all subjects, if the minimum k-anonymity for any point is < 10, the perturbation is not considered to meet HIPAA de-identification standards. Because the k-anonymity provided by a perturbation is a function of the spatial density of the data, we performed perturbations on both the full dataset as well as down-sampled data, e.g. randomly sampling only 75% of the available points, to understand the impact of the density of the unperturbed data on the degree of anonymity conferred by each approach (Fig. 7).

## Results

**Fig. 8:**
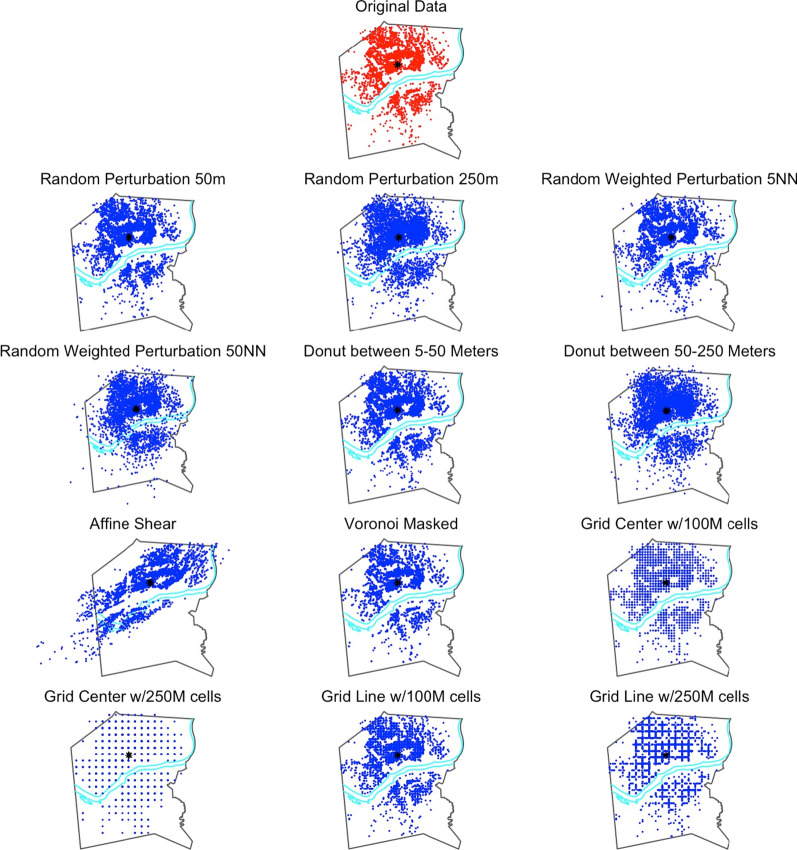
Each of the data perturbations applied to the Lawrence, MA data. The center of the spatial distribution is marked by a black star. The light blue line represents the Merrimack River, which cuts through Lawrence

In this section, we review the impact of each of the different approaches to perturbation outlined above on the spatial characteristics of the perturbed datasets, as well as the degree of anonymization conferred by each approach. Maps of illustrating the impact of the perturbation on the mortality data are available in Appendix (Fig. 8).

### Impact of perturbation on key spatial statistics

- Point center: Affine shear moved the median of the spatial distribution the farthest Euclidean distance, followed by grid center masking with 100 × 100 m cells, and grid center masking with 250 × 250 m cells, which moved the median 123 m, 42 m, and 33 m, respectively. All other perturbations had little effect, moving the median spatial center less than 10 m in Euclidean distance; additionally, none of the perturbations moved the mean center of the spatial distribution more than 5 m in Euclidian distance. The effects of each perturbation can be seen in Table 1.

**Table 1:** Euclidean distance between the original spatial center of the distribution, as depicted by both median and mean of the points

Perturbation method	Change in median	Change in mean
Original data	0	0
Random perturbation 50 m	0.32	0.11
Random perturbation 250 m	0.79	0.82
Random perturbation 500 m	18.54	0.63
Random weighted perturbation 5 NN	1.76	0.35
Random weighted perturbation 50 NN	3.69	1.09
Donut between 5 and 50 m	3.76	0.28
Donut between 50 and 250 m	2.65	5.58
Affine shear	123.49	0.38
Voronoi masked	0.84	0.18
Grid center w/100 m cells	42.09	1.6
Grid center w/250 m cells	32.91	4.29
Grid line w/100 m cells	7.91	0.6
Grid line w/250 m cells	6.68	1.29
- Global and Local Moran’s I: When aggregating points to 200 × 200 m cells, the unperturbed data had a Global Moran’s I of 0.58, indicating positive spatial autocorrelation between the numbers of deaths in each cell. Although all the perturbations maintained a positive value of Global Moran’s I, grid center masking with both 100 × 100 m and 250 × 250 m cells resulted in greatly decreased values of *I*, from 0.58 to 0.36 and 0.30, respectively. Grid line masking with 250 × 250 m cells and random weighted perturbation within the 5th nearest neighbor also decreased the Global Moran’s I to 0.52 and 0.55, respectively. All other perturbations increased the *I* value, with donut masking between 50 and 250 m increasing the value the most to 0.79. The effects of all perturbations can be seen in Table 2. Trends of local Moran’s I were similar. Choropleths of Local Moran’s I demonstrate the change in spatial autocorrelation for the number of deaths per 200 × 200 m cells and can been viewed in Fig. 9 of Appendix.

**Table 2:** Global Moran’s I statistic for each of the perturbation methods

Perturbation method	Global Moran’s I	Proportion of unperturbed I	Variance in I
Original data	0.49	1	0.49302
Random perturbation 50 m	0.54	0.91	0.54023
Random perturbation 250 m	0.82	0.6	0.82067
Random perturbation 500 m	0.89	0.55	0.88739
Random weighted perturbation 5 NN	0.57	0.86	0.56697
Random weighted perturbation 50 NN	0.58	0.84	0.57983
Donut between 5 and 50 m	0.55	0.89	0.54593
Donut between 50 and 250 m	0.8	0.61	0.80124
Affine shear	0.54	0.91	0.53597
Voronoi masked	0.48	1.02	0.48222
Grid center w/100 m cells	0.36	1.36	0.35639
Grid center w/250 m cells	0.3	1.63	0.30335
Grid line w/100 m cells	0.47	1.04	0.47285
Grid line w/250 m cells	0.44	1.11	0.43534
- Distance to kth-Nearest Neighbor: For each perturbation, as *k* increased, the average distance to the k-th nearest neighbor became more similar to the distances for the unperturbed data. Aggregating perturbations decreased the average distance at all values of k, while non-aggregating perturbations increased the distance to the k-th nearest neighbor. Voronoi masking, which has both aggregating and non-aggregating properties because some points are moved together, greatly decreased the average distance to the 1st nearest and neighbor but maintained the average distance to all other neighbors (Table 3).

**Table 3:** Distance to different nearest neighbors compared to the unperturbed data

Perturbation method	K = 1	Proportion of original K = 1	K = 5	Proportion of original K = 5	K = 10	Proportion of original K = 10	K = 20	Proportion of original K = 20
Original data	14.57	1	47.67	1	71.42	1	104.09	1
Random perturbation 50 m	20.52	1.41	52.19	1.09	74.28	1.04	106.41	1.02
Random perturbation 250 m	25.07	1.72	62.03	1.3	87.37	1.22	122.83	1.18
Random perturbation 500 m	27.2	1.87	67.12	1.41	95.37	1.34	134.57	1.29
Random weighted perturbation 5 NN	20.38	1.4	48.54	1.02	71.65	1	104.73	1.01
Random weighted perturbation 50 NN	25.02	1.72	60.06	1.26	85.36	1.2	118.45	1.14
Donut between 5 and 50 m	20.5	1.41	52	1.09	74.45	1.04	106.31	1.02
Donut between 50 and 250 m	24.95	1.71	61.28	1.29	86.8	1.22	122.93	1.18
Affine shear	15.2	1.04	48.6	1.02	73.03	1.02	106.97	1.03
Voronoi masked	5.59	0.38	46.38	0.97	70.77	0.99	103.29	0.99
Grid center w/100 m cells	5.94	0.41	34.79	0.73	62.48	0.87	97.84	0.94
Grid center w/250 m cells	1.53	0.11	12.82	0.27	29.1	0.41	62.89	0.6
Grid line w/100 m cells	11.39	0.78	44.58	0.94	69.86	0.98	104.75	1.01
Grid line w/250 m cells	7.31	0.5	32.94	0.69	58.26	0.82	94.25	0.91
- L-Function: To understand the impact of each perturbation on the spatial dispersion of points, the L-function was measured for each perturbation and compared to the original data. Voronoi masking had the least effect on the L-function, while affine shear and grid center masking at both 100- and 250-m cells had the greatest. Results can be seen in Fig. 10 of Appendix.

### Impact of perturbation on data privacy

Using the complete dataset, there was no perturbation that met the HIPAA standard of including no points with k-anonymity < 10. For clarity, we denote k-anonymity as $\rho$ and average k-anonymity as $\overline{\rho }$. Affine shearing provided the greatest privacy protection, with 265 cases (6.5%) with $\rho \, < \,10$ and 134 cases (3.3% of cases) with $\rho \, < \,5.$ Grid center masking with 250 m^2^ cells resulted in 357 cases (8.8% of cases) with $\rho \, < \,10$ and 159 (3.9% of cases) with $\rho \, < \,5$. All other approaches left at least 623 cases (or 15.4% of all cases) with $\rho$ < 10. Voronoi masking conferred the least anonymity, with $\overline{\rho }\, =$ 1.90 and all points having $\rho$ < 10. When using a random sample of 75% of the cases, none of the perturbations met the HIPAA standard of all points having a k-anonymity greater than or equal to 10. As the percent of points released decreases, anonymity for those points also fell, underscoring how high spatial density increases individual privacy when measured using k-anonymity. K-anonymity for all perturbations with multiple sub-samples of the data are presented in Table 4 in Appendix.

Taken together, our results indicate that obtaining the level of de-identification required by HIPAA, GDPR and similar regulatory standards using the perturbation methods we employed, significant alterations of some key spatial patterns were required. Affine shearing provided the greatest K-anonymity but had large impacts on the spatial center of the distribution and strongly altered patterns of Local Moran’s I. Grid-center masking with 250-m cells provides the next greatest K-anonymity, but also significantly alters values of key statistics, including global and local Moran’s I, and Ripley’s K/L.

## Discussion

Our results show that the wide range of perturbation methods applied in this analysis were not compatible with HIPAA and GDPR-compliant de-identification when the results also maintained key spatial patterns as measured by the chosen summary statistics. This highlights the significant challenge of safely releasing spatial health datasets while preserving enough information content to make them useful for analysis. Affine shear conferred the greatest anonymity using the k-anonymity metric and maintained some spatial patterns. However, the method is not secure, as points can be trivially re-identified if the angle of the shearing can be determined. Spatial features, such as the Merrimack River in this dataset, would indicate where the true locations of cases could not be, and reverse-engineering around these and other geographic features could then easily be undone to obtain the shearing angle. Grid center masking with cells of 250 × 250 m resulted in large changes in global Moran’s I values and dramatically altered the distribution of local clustering indicators (e.g. local Moran’s I) but also provided the greatest de-identification as measured by k-anonymity that is not as vulnerable to reverse engineering as easily as affine shearing. However, grid center masking with cells of 250 × 250 m still did not meet HIPAA standards for privacy (minimum $\rho \, \ge$ 10 for the entire dataset) with 357 cases with $\rho \, <$ 10.

Voronoi masking, random perturbation, and random weighted perturbation had the smallest impact on the original spatial patterns, but also provided minimal de-identification, with hundreds of points having $\rho$ < 10 and a minimum $\rho$ = 1. Voronoi masking was either the first or second closest to the original value for all measures of spatial aggregation, indicating that while unaltered Voronoi masking may not provide de-identification thorough enough to meet HIPAA standards, it does maintain underlying spatial patterns better than other methods of geomasking. This suggests that efforts to build on Voronoi-based approaches may be fruitful. For example, using multiple iterations of the Voronoi tessellation algorithm, known as Lloyd’s algorithm, as well as combining a stochastic perturbation technique with Voronoi masking [33]. Another possibility is to take an iterative approach to maximizing k-anonymity, e.g. by applying a stronger perturbation to individuals $\rho$ < 10 after the first application of an approach that works well and provides $\rho$ ≥ 10 for the large majority of points.

Although closer to the regulatory standards than all other perturbations except affine shear, grid center masking with cells of 250 × 250 m strongly degraded all of the spatial measures employed. Because grid center masking is an aggregating perturbation, it decreased the distance to kth-nearest neighbors as well as Global Moran’s I. Although grid center masking with such large cells may not provide high fidelity for spatial statistics at the fine scale examined here, the deterministic nature of the of perturbation results in predictable biases of the underlying statistics. A further analysis of these relationships may be helpful for estimating correction factors that can be used to adjust estimates derived from perturbed data so that they are closer to those derived from the underlying data.

Our analysis has a number of strengths. Unlike previous research, the anonymity metric used to measure de-identification was specifically derived from the HIPAA standard and also meets GDPR standards. This provides a realistic measure of the likelihood that a given approach will produce outputs that accord with global health privacy laws. Additionally, our direct comparisons of a variety of perturbation measures using a single policy-relevant anonymization metric may aid in the development of a consensus around how and when these different approaches should be applied.

Despite these strengths, these results also have several important limitations. For example, they are limited by the use of a single spatial dataset characterized by strong spatial clustering representative of data from a densely populated urban neighborhood or small city. The lack of data about surrounding non-case households also prevented the use of some advanced geomasking techniques [34–36]. It is also unavoidable that different perturbations will have different implications when the underlying data have different spatial characteristics, e.g. the presence of multiple distinct spatial clusters, lower density of points over a larger spatial area, etc. In addition, the original mortality data demonstrated significant spatial autocorrelation with a statistically significant Global Moran’s I of 0.58. Because aggregating perturbations will always move points together and create empty spaces where points previously were, they will always bias Moran’s I towards greater dispersion given the true underlying distribution. If the true data were less clustered, aggregating methods of perturbation might produce different biases. An important next step towards developing a set of broadly-applicable best practices for privacy-first reproducibility is performing the analyses presented here on datasets characterized by different densities and spatial scales. Future studies should investigate the effect that differences in the underlying data have on the tradeoff between de-identification and maintenance of spatial patterns.

Despite its broad use as a measure of spatial anonymity, k-anonymity may in fact not be ideal for this purpose. For example, in the context of non-spatial data, ensuring that an individual cannot be distinguished from *k* other individuals in the same dataset may be reasonable. Although this dataset allowed for a realistic examination of anonymity when only cases are geocoded, additional information about the background population would allow for a different interpretation of k-anonymity. However, k-anonymity for spatial data is heavily influenced by the point density of the original data: if points are very close together, the k-anonymity conferred by a perturbation may be large even though the actual distance between the original and perturbed locations is very small. The risk posed to privacy becomes clear when other sources of spatial population data are available, e.g. from census data or via projects such as WorldPop [37]. This means that individuals not included in the original dataset may be at risk of identification when spatial data and key publicly available metadata elements are linked (e.g. population density, age distributions, race/ethnicity, sex/gender breakdowns). Consequently, even if a perturbation increases within-dataset anonymity, it may have little to no impact on privacy at the population level if it provides information on risk in the underlying population that can be extracted via approaches such as a kriging and other methods of spatial interpolation and smoothing.

Future studies should investigate alternative approaches to spatial de-identification that address the limitations of within-dataset k-anonymity discussed here. More advanced geomasking techniques exist that require additional information about surrounding households; location swapping, the verified neighbor approach, and adaptive aerial elimination may provide greater anonymity but also require extensive spatial information about the region. These methods require not only the locations of cases, but also the centroids of surrounding households which are not always available, such as with our dataset [34–36]. In addition, these questions become more complex when additional information beyond the spatial location of a case is included in a dataset, e.g. age, sex, comorbidity status, etc.

## Conclusions

Resolving the technical, ethical and legal issues surrounding spatial data anonymization will have positive benefits for researchers, patients, and policymakers across the health sciences. The urgency of these questions is clear: as the response to COVID-19 has shown, high-resolution data can be helpful for informing both short-term tactics and long-term strategies in public health response [38, 39]. But the benefits of more granular public data will not be realized if individual privacy cannot be reliably protected. For such tools to be useful in future emergencies, a well-defined and agreed-upon set of privacy and technical standards for anonymization must be available so that they can be rapidly deployed while meeting ethical and legal standards.

Although we used HIPAA as a benchmark, the approaches described here have clear relevance to other types of data not subject to HIPAA protection, but for which ethical and legal barriers to full reproducibility still exist. For example, effective intervention to prevent human trafficking and other forms of exploitation may aided by geospatial data, while the underlying location of reported events is clearly sensitive and may be legally protected in some jurisdictions, e.g. under GDPR rules.

Ultimately, there are no one-size-fits-all solutions to the problem of spatial data anonymization. Instead, open-source software employing validated approaches to secure data anonymization are necessary to attain the balance of anonymization and fidelity necessary to meet privacy standards while maintaining utility for the intended application. Our analysis represents a step towards achieving these goals. However, further research focused on facilitating openness and reproducibility while complying with ethical and legal standards is sorely needed to advance the impact of the spatial sciences across public health, medicine, and the social sciences.

## Figures and Tables

**Fig. 9:**
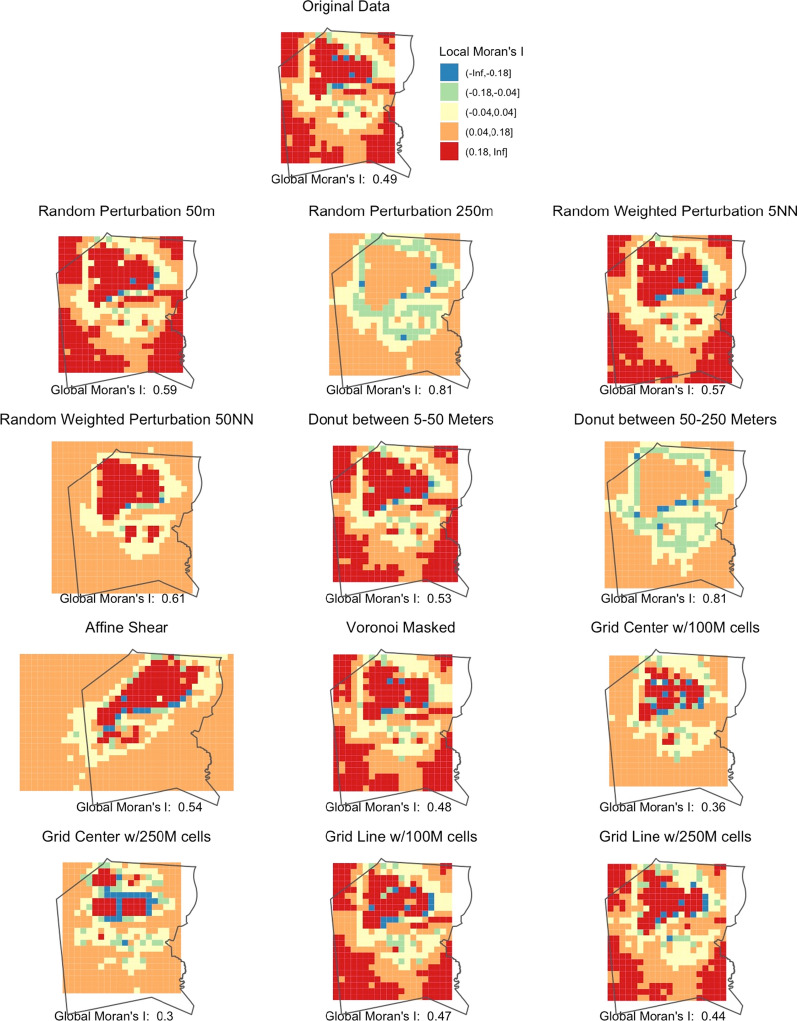
Choropleths of local Moran’s I for each perturbation. The global Moran’s I is listed underneath

**Fig. 10:**
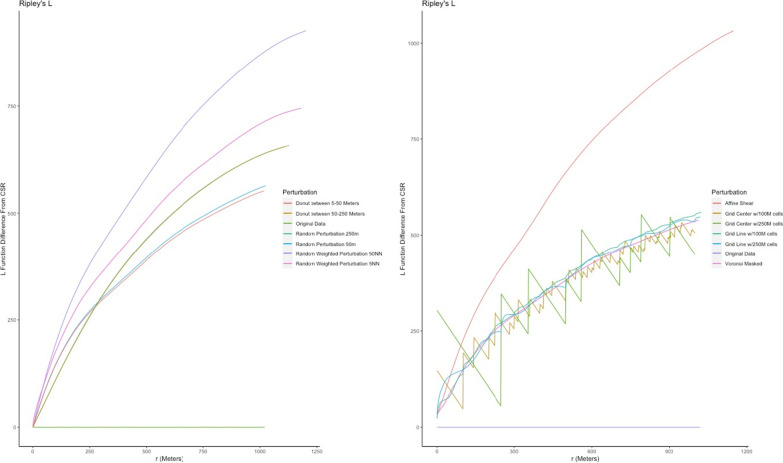
The original data was centered at zero so each perturbation’s function shows the increased divergence from complete spatial randomness beyond the original data

## Data Availability

Data and code are available at https://github.com/broenk/Spatial_Perturbation.

## Appendix

See Figs. 8, 9, 10 and Table 4.

**Table 4:** K-anonymity for each perturbation and with different amounts of data release

Perturbation method	100% of data					75% of data					50% of data				
	Points per km^2^	K < 10	K < 5	Min K	Mean K	Points per km^2^	K < 10	K < 5	Min K	Mean K	Points per km^2^	K < 10	K < 5	Min K	Mean K
Original data	211.27	1	0.9938	1	1.3699	158.48	1	1	1	1.2745	105.63	1	1	1	1.1798
Random perturbation 50 m	211.27	0.7509	0.5395	1	6.9993	158.48	0.8183	0.6093	1	5.4984	105.63	0.9042	0.7057	1	3.9531
Random perturbation 250 m	211.27	0.1309	0.0644	1	77.2057	158.48	0.1669	0.0839	1	58.9595	105.63	0.2267	0.1146	1	38.7753
Random perturbation 500 m	211.27	0.0415	0.0173	1	219.025	158.48	0.0523	0.0263	1	166.9783	105.63	0.078	0.0365	1	110.491
Random weighted perturbation 5 NN	211.27	0.9672	0.7738	1	3.3728	158.48	0.9839	0.8515	1	2.7956	105.63	0.9921	0.9328	1	2.1738
Random weighted perturbation 50 NN	211.27	0.2326	0.0928	1	26.1316	158.48	0.3041	0.132	1	19.9167	105.63	0.4385	0.1951	1	14.0489
Donut between 5 and 50 m	211.27	0.7565	0.5323	1	6.9296	158.48	0.8209	0.6017	1	5.4299	105.63	0.9111	0.7195	1	3.9086
Donut between 50 and 250 m	211.27	0.1015	0.042	1	78.3049	158.48	0.1389	0.0579	1	60.0471	105.63	0.2084	0.0928	1	39.7427
Affine shear	211.27	0.0654	0.0331	1	537.758	158.48	0.0787	0.0395	1	407.0853	105.63	0.1012	0.0553	1	272.196
Voronoi masked	211.27	1	0.9911	1	1.902	158.48	1	0.999	1	1.681	105.63	1	1	1	1.4538
Grid center w/100 m cells	211.27	0.4852	0.2309	1	16.3402	158.48	0.5329	0.2956	1	12.7643	105.63	0.6405	0.4227	1	8.5289
Grid center w/250 m cells	211.27	0.0881	0.0393	1	66.0889	158.48	0.1172	0.055	1	50.1178	105.63	0.1936	0.0894	1	33.7496
Grid line w/100 m cells	211.27	0.8333	0.636	1	5.3649	158.48	0.8756	0.7143	1	4.2074	105.63	0.9467	0.7975	1	3.1274
Grid line w/250 m cells	211.27	0.4743	0.2859	1	19.6706	158.48	0.5467	0.3377	1	15.1968	105.63	0.6563	0.4163	1	10.1891

Perturbation method	25% of data					10% of data				
	Points per km^2^	K < 10	K < 5	Min K	Mean K	Points per km^2^	K < 10	K < 5	Min K	Mean K
Original data	52.79	1	1	1	1.081	21.13	1	1	1	1.0346
Random perturbation 50 m	52.79	0.9901	0.8913	1	2.3241	21.13	1	0.963	1	1.6296
Random perturbation 250 m	52.79	0.3893	0.2243	1	19.2826	21.13	0.6321	0.3877	1	9.3901
Random perturbation 500 m	52.79	0.1492	0.0731	1	56.6166	21.13	0.2988	0.1728	1	22.8
Random weighted perturbation 5 NN	52.79	0.999	0.9832	1	1.6136	21.13	1	0.9951	1	1.2864
Random weighted perturbation 50 NN	52.79	0.7658	0.4071	1	7.1897	21.13	0.958	0.7432	1	3.5259
Donut between 5 and 50 m	52.79	0.9881	0.8498	1	2.4496	21.13	1	0.9704	1	1.6074
Donut between 50 and 250 m	52.79	0.3607	0.167	1	19.3142	21.13	0.6222	0.3506	1	9.6173
Affine shear	52.79	0.1542	0.0939	1	136.2579	21.13	0.2049	0.1432	1	53.4469
Voronoi masked	52.79	1	1	1	1.2036	21.13	1	1	1	1.1111
Grid center w/100 m cells	52.79	0.8794	0.6077	1	4.6285	21.13	0.9753	0.8272	1	2.7086
Grid center w/250 m cells	52.79	0.3745	0.1611	1	16.5237	21.13	0.7062	0.3679	1	7.9877
Grid line w/100 m cells	52.79	0.999	0.9328	1	1.9713	21.13	1	0.9827	1	1.4889
Grid line w/250 m cells	52.79	0.83	0.6047	1	5.4229	21.13	0.9506	0.7975	1	3.0469
